# A Multifunctional γ-Polyglutamic Acid Hydrogel for Combined Tumor Photothermal and Chemotherapy

**DOI:** 10.3390/gels11030217

**Published:** 2025-03-20

**Authors:** Xiaoqing Jia, Shige Wang

**Affiliations:** School of Materials and Chemistry, University of Shanghai for Science and Technology, No. 516 Jungong Road, Shanghai 200093, China; 221600226@st.usst.edu.cn

**Keywords:** photothermal therapy, hydrogel, tumor treatment, γ-polyglutamic acid, drug delivery

## Abstract

Efficient and precise cancer therapy remains a challenge due to limitations in current treatment modalities. In this study, we developed a multifunctional hydrogel system that integrates photothermal therapy (PTT) and chemotherapy to achieve combined tumor treatment. The hydrogel, composed of γ-polyglutamic acid (γ-PGA), fifth-generation polyamide-amine dendrimers (G5), and polydopamine (PDA) nanoparticles, exhibits high photothermal conversion efficiency and temperature-responsive drug release properties. The hydrogel exhibited a high photothermal conversion efficiency of 45.6% under 808 nm near-infrared (NIR) irradiation. Drug release studies demonstrated a cumulative hydrophilic anticancer drug doxorubicin DOX release of 79.27% within 72 h under mild hyperthermia conditions (50 °C). In vivo experiments revealed a significant tumor inhibition rate of 82.3% with minimal systemic toxicity. Comprehensive in vitro and in vivo evaluations reveal that the hydrogel demonstrates excellent biocompatibility, photothermal stability, and biodegradability. Unlike conventional hydrogel systems, our γ-PGA-based hydrogel uniquely integrates a biocompatible and biodegradable polymer with polydopamine (PDA) nanoparticles, providing a smart and responsive platform for precise cancer therapy. This multifunctional hydrogel system represents a promising platform that combines PTT precision and chemotherapy efficacy, providing a robust strategy for advanced and safer cancer treatment.

## 1. Introduction

Cancer therapy remains an enduring challenge due to the limitations of conventional treatment modalities, including surgery, radiotherapy, and chemotherapy [1,2,3,4,5]. While these approaches have achieved notable clinical success, they are often associated with significant drawbacks, such as non-specific cytotoxicity, incomplete tumor ablation, and severe side effects that compromise the therapeutic outcomes. Therefore, there is a pressing need for the development of novel therapeutic strategies that combine precision, efficiency, and minimal invasiveness to enhance cancer treatment efficacy. Photothermal therapy (PTT) has emerged as a transformative approach to cancer treatment, offering distinct advantages over conventional methods [6,7,8]. By using lasers of specific wavelengths to activate photothermal agents, PTT induces localized hyperthermia, effectively ablating tumor cells with minimal invasiveness [9,10]. This approach exhibits high specificity, controllability, and reduced collateral damage to surrounding healthy tissues. However, PTT faces challenges, such as limited penetration depth of laser light and uneven heat distribution, which constrain its efficacy, particularly in treating heterogeneous or deep-seated tumors [11,12].

To address these limitations, combined therapeutic strategies that combine PTT with chemotherapy have gained research interest [13,14]. This dual-modality approach leverages the localized thermal effects of PTT to enhance the sensitivity of tumor cells to chemotherapeutic agents while enabling temperature-controlled drug release. By integrating the precision of PTT with the systemic therapeutic effects of chemotherapy, these strategies can achieve enhanced tumor ablation at reduced drug dosages, minimizing systemic toxicity [15,16]. Therefore, the combined effects of PTT and chemotherapy hold promise for overcoming the intrinsic limitations of each modality, offering a robust and versatile framework for cancer therapy.

Hydrogels, as a class of soft materials with three-dimensional network structures, are gaining attention for their applications in drug delivery and tumor therapy [17,18]. With high water content and excellent biocompatibility, hydrogels can provide a favorable microenvironment for therapeutic interventions [19,20,21]. Their tunable porous structures allow precise control over drug loading and release kinetics, while their responsiveness to external stimuli, such as temperature, pH, and light, further enhances their utility as multifunctional therapeutic platforms [22,23,24]. Moreover, their capacity to encapsulate bioactive compounds and photothermal agents makes them ideal candidates for combined therapeutic strategies [25,26,27]. Recent advancements have explored the incorporation of photothermal agents, such as PDA nanoparticles, into hydrogels to achieve combined therapeutic outcomes [28]. These integrations not only enhance the photothermal conversion efficiency but also enable controlled and localized drug delivery, addressing key limitations in conventional treatment methods. Compared to conventional hydrogel matrices such as alginate or chitosan, γ-PGA offers superior water solubility, controlled biodegradability, and pH-responsive behavior [29], making it highly suitable for tumor-targeted drug delivery. Its anionic nature also allows for efficient interaction with cationic therapeutic agents, enhancing drug retention and release profiles [30,31].

In this study, we developed a novel multifunctional hydrogel incorporating γ-polyglutamic acid (γ-PGA), fifth-generation dendrimers (G5), and PDA nanoparticles. This hydrogel system integrates photothermal conversion and controlled drug release, enabling combined PTT and chemotherapy. The hydrogel leverages the amino groups of G5 to facilitate the polymerization of dopamine into PDA nanoparticles, which are subsequently embedded in the hydrogel matrix through the chemical cross-linking between amino groups of G5 and carboxyl of γ-PGA. Before the hydrogel formation, DOX was first dissolved in the γ-PGA solution, enabling direct drug encapsulation. This unique structure allows precise control over the release kinetics of encapsulated drugs, while the PDA nanoparticles provide robust photothermal conversion under near-infrared (NIR) laser. The resulting system exhibits excellent photothermal responsiveness, controlled degradation, and sustained drug release capabilities, making it a versatile platform for cancer treatment. Systematic evaluations of the hydrogel demonstrate its biocompatibility, efficient photothermal conversion, and significant tumor cell ablation both in vitro and in vivo. In vitro studies revealed rapid temperature increases under laser irradiation, facilitating targeted drug release and tumor cell apoptosis. In vivo experiments further validated the hydrogel’s therapeutic efficacy, showcasing pronounced tumor suppression with minimal systemic toxicity. Additionally, the hydrogel’s biodegradability eliminates the need for secondary removal post-treatment, simplifying clinical applications and reducing patient risk. Our findings highlight the potential of this multifunctional hydrogel as a next-generation therapeutic platform, combining the precision of PTT with the systemic benefits of chemotherapy. Therefore, this study aims to develop a γ-PGA-based hydrogel system that integrates photothermal therapy (PTT) and chemotherapy for improved cancer treatment. The novelty of this system lies in its dual-responsive nature, where NIR irradiation not only induces localized hyperthermia but also enhances drug release in a controlled manner.

## 2. Results and Discussion

### 2.1. Synthesis and Characterization of Hydrogel

**Figure 1:**
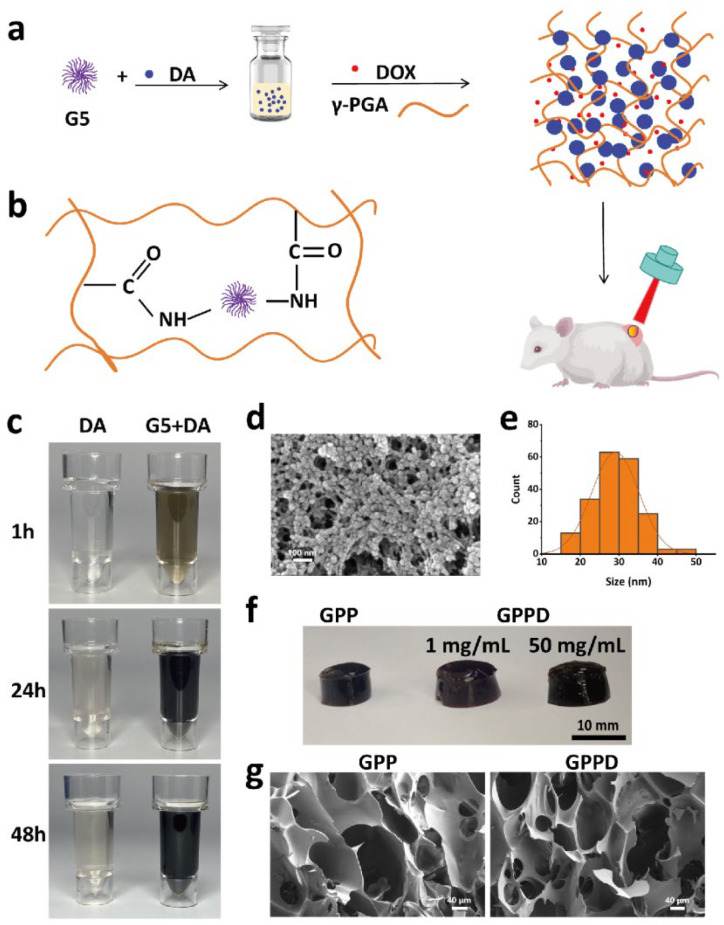
(**a**) Schematic illustration of the material design and tumor therapy applications; (**b**) diagram showing the internal bonding structure of the hydrogel; (**c**) schematic illustration of PDA formation from dopamine hydrochloride catalyzed in the presence of G5; (**d**) SEM image of PDA nanoparticles; (**e**) size distribution of PDA nanoparticles; (**f**) photographs of GPP and GPPD hydrogels; and (**g**) SEM images of GPP and GPPD hydrogels.

The design, synthesis, and structural features of the hydrogel system are schematically depicted in Figure 1a,b. Initially, dopamine hydrochloride was polymerized to form PDA nanoparticles with excellent photothermal conversion capability in the presence of G5 dendrimer. Subsequently, the carboxyl groups on the γ-PGA chains were activated by 1-ethyl-3-(3-dimethylaminopropyl) carbodiimide hydrochloride (EDC), and N-hydroxysuccinimide (NHS), enabling rapid covalent cross-linking with the amino groups on G5 dendrimers. This process resulted in the formation of the GPP hydrogel within 1 min. The resulting hydrogel exhibits excellent photothermal responsiveness, controlled degradation, and sustained drug release capabilities, making it a versatile platform for cancer treatment.

As illustrated in Figure 1c, the dopamine solution immediately darkened upon the addition of G5, which facilitated dopamine polymerization into black PDA nanoparticles. In contrast, the control solution without G5 exhibited a much slower color change over 48 h, confirming the critical role of G5 in the polymerization process. The morphology of the synthesized PDA nanoparticles was further examined using SEM (Figure 1d). The nanoparticles displayed a spherical shape with a uniform size distribution, demonstrating the successful polymerization and stabilization of PDA. PDA nanoparticles exhibited a mean diameter of 27.5 nm as confirmed by SEM. To evaluate the hydrogel’s versatility for drug delivery, the hydrophilic anticancer DOX was introduced during the precursor mixing stage. As depicted in Figure 1e, the GPP hydrogel retained its excellent gelation properties even at high drug concentrations. The gelation state of the DOX-loaded hydrogel (GPPD) was maintained, with a broad range of drug-loading capacities reaching up to 50 mg/mL, which is advantageous for tailored therapeutic applications.

Following gelation, both GPP and GPPD hydrogels were freeze-dried, and their internal microstructure was analyzed via SEM. As shown in Figure 1f, the hydrogels exhibited a highly porous and interconnected three-dimensional structure. These porous networks not only enhance water uptake and biocompatibility but also provide an optimal microenvironment for efficient drug encapsulation and sustained release. The successful integration of PDA nanoparticles and DOX within the hydrogel matrix demonstrates its structural stability and suitability for multifunctional therapeutic applications.

### 2.2. Rheological Characteristics and Swelling of Hydrogels

**Figure 2:**
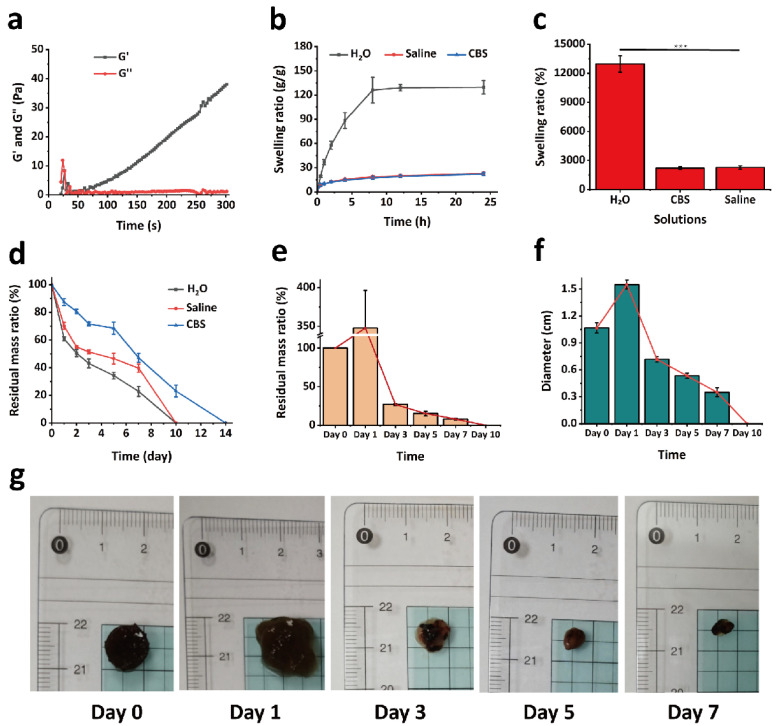
(**a**) Rheological behavior of GPPD hydrogel; (**b**) swelling kinetics of GPPD hydrogel in various solutions; (**c**) 24 h swelling rates of GPPD hydrogel in different solutions (*** *p* < 0.001); (**d**) in vitro degradation profile of GPPD hydrogel; (**e**,**f**) in vivo degradation of GPP hydrogel (**e**): weight changes; (**f**): size changes); and (**g**) representative in vivo images of GPP hydrogel during the degradation.

The gelation kinetics and mechanical properties of the GPPD hydrogels were evaluated through rheological testing to monitor their transition from sol to gel states. As shown in Figure 2a, upon the addition of the EDC/NHS activator, the G′ rapidly increased and surpassed the G″ within approximately 40 s. This significant rise in G′ indicates the formation of a stable cross-linked network structure, which reflects the efficient covalent bonding between the γ-PGA and G5 dendrimer molecules. Over time, G′ continued to increase until reaching a plateau, confirming the complete gelation of the hydrogel system. These results demonstrate that the hydrogel forms quickly, providing structural stability essential for biomedical applications.

The swelling behavior of GPPD hydrogel was systematically evaluated under different environmental conditions to simulate physiological and tumor microenvironments (Figure 2b,c). The hydrogel exhibited remarkable swelling performance in deionized water, achieving an equilibrium swelling ratio of 12,958.81 ± 849.47% after 24 h. In saline and citrate buffer solution (CBS) (pH 5.4), the swelling ratios were significantly lower, reaching 2272.39 ± 177.48% and 2206.18 ± 144.61% (*** *p* < 0.001 versus group H_2_O), respectively. This difference can be attributed to the high ionic concentration in saline and acidic environments, which increases osmotic pressure and reduces the water uptake capacity of the hydrogel. The presence of carboxyl (-COOH) groups on γ-PGA chains enhances the hydrogel’s hydrophilicity through hydrogen bonding with water molecules, promoting its swelling capacity. The reduced swelling in physiological and acidic solutions, however, ensures the hydrogel maintains its structural integrity under biologically relevant conditions. The controlled swelling behavior of the hydrogel in physiological conditions enhances its structural stability, ensuring sustained drug release and prolonged retention at the tumor site. These swelling characteristics make GPPD hydrogel particularly suitable for drug delivery systems, where controlled hydration and stability are critical for sustained release.

### 2.3. Degradation of Hydrogels In Vitro and In Vivo

The degradation behavior of the GPPD hydrogel was thoroughly investigated both in vitro and in vivo to assess its stability and suitability for biomedical applications. Controlled degradation is crucial for achieving sustained drug release and minimizing the need for post-treatment surgical removal. As shown in Figure 2d, the in vitro degradation profiles of the hydrogels in deionized water, saline, and CBS buffer indicate that the materials degraded completely within 14 days. In the acidic environment (CBS), the degradation rate was slightly slower compared to neutral conditions, likely due to the reduced hydrolytic activity under acidic pH. Nevertheless, the material exhibited consistent degradation behavior across all tested environments.

The in vivo degradation of GPP hydrogel was evaluated by subcutaneous implantation in KM mice (Figure 2e–g). Upon implantation, the hydrogel rapidly absorbed body fluids, causing significant swelling within the first day. The mass of the hydrogel decreased to 26.91% after 3 days and further reduced to 15.30% by day 5. By day 10, the hydrogel had completely degraded, demonstrating a faster degradation rate in vivo compared to in vitro conditions. This accelerated degradation is attributed to the enzymatic activity and complex physiological environment in vivo, which promote the breakdown of the hydrogel’s chemical bonds. Hydrogel degradation occurs via hydrolysis of γ-PGA ester bonds, leading to gradual mass loss. In vivo studies confirmed a faster degradation rate compared to in vitro conditions. Visual observations (Figure 2g) confirmed the gradual reduction in hydrogel size over time, indicating its effective biodegradability. The observed degradation rate ensures sustained drug release while eliminating the need for secondary removal after treatment, thus simplifying clinical applications and reducing patient burden, showing significant advantages over other non-degradable drug carriers. These results highlight the hydrogel’s excellent balance between structural stability and controlled degradation, making it a promising candidate for drug delivery and therapeutic applications in vivo.

### 2.4. In Vitro Photothermal Conversion

**Figure 3:**
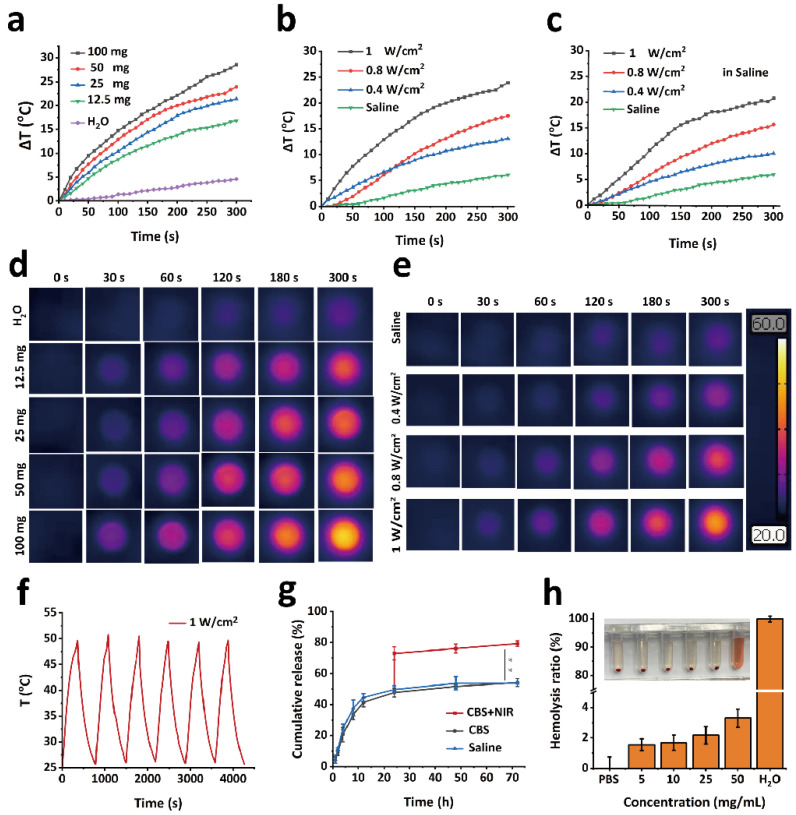
(**a**,**b**) Temperature change curves of GPPD hydrogels under NIR irradiation with varying masses and laser powers; (**c**) temperature change in physiological saline; (**d**,**e**) thermal imaging of GPPD hydrogels with different masses and laser powers under NIR irradiation; (**f**) thermal cycling stability; (**g**) in vitro DOX release from GPPD hydrogel (** *p* < 0.01); and (**h**) hemocompatibility results of GPPD hydrogel.

The photothermal properties of the GPPD hydrogel were systematically evaluated to determine its efficacy under NIR irradiation. First, GPPD hydrogels of varying masses (12.5, 25, 50, and 100 mg) were irradiated with an 808 nm laser at a fixed power density of 1 W/cm^2^ for 5 min (Figure 3a). The temperature rise, recorded using an infrared thermal imaging camera, revealed a positive correlation between hydrogel mass and temperature elevation, with the 100 mg hydrogel exhibiting the highest temperature increase of 28.6 °C. In contrast, the control group (deionized water) displayed minimal thermal changes under identical conditions, confirming the hydrogels’ superior photothermal responsiveness. Then, the effect of laser power was assessed by irradiating a fixed mass (50 mg) of GPPD hydrogel with laser intensities of 0.4, 0.8, and 1 W/cm^2^ (Figure 3b). The results demonstrated a proportional increase in temperature with higher power densities, further validating the hydrogel’s efficient photothermal conversion capabilities. In addition, photothermal performance in a physiological saline environment was evaluated to simulate in vivo conditions. Under saline immersion, the hydrogels maintained comparable photothermal performance (Figure 3c), indicating their applicability in biological environments. Thermal imaging photographs (Figure 3d) further qualitatively prove the good photothermal performance of GPPD hydrogel. To assess thermal stability, GPPD hydrogels were subjected to six consecutive heating–cooling cycles under NIR irradiation at 1 W/cm^2^. The temperature profiles showed negligible attenuation across all cycles (Figure 3f), confirming the hydrogels’ robust photothermal stability and reusability. Together, these results highlight the hydrogel’s excellent photothermal properties, stability, and responsiveness, which are essential for its application in precision cancer treatment.

### 2.5. Drug Loading and Drug Release of Hydrogel

To investigate the sustained drug release performance of the drug-loaded hydrogel system, a series of controlled-release experiments were conducted. Drug release kinetics were evaluated in both physiological saline and citrate buffer solution (CBS, pH 5.4) to simulate normal and tumor microenvironments, respectively (Figure 3g). Under normal conditions, the cumulative release of DOX showed no significant differences between physiological saline (54.23%) and acidic CBS (53.75%) environments within the first 72 h. However, upon exposure to elevated temperatures, mimicking photothermal stimulation, a pronounced burst release of DOX was observed in the CBS group (79.27%). Specifically, subjecting the hydrogel to 50 °C for 5 min resulted in a significant increase in the drug release rate (** *p* < 0.01 versus group CBS). This rapid release phenomenon can be attributed to the temperature-induced collapse and accelerated degradation of the hydrogel network, which facilitated enhanced diffusion of DOX molecules from the hydrogel matrix. The pH-dependent behavior of the GPPD hydrogel under laser irradiation can be attributed to the protonation of carboxyl groups on the γ-PGA chains in acidic conditions, which is typical of the tumor microenvironment (pH ~5.4). Under acidic conditions, the carboxyl groups (-COOH) on γ-PGA become partially protonated, leading to changes in the hydrogel’s swelling behavior and network structure. This protonation reduces the electrostatic repulsion between the polymer chains, causing the hydrogel to swell less compared to neutral conditions. However, when combined with laser irradiation, the localized heating from the photothermal effect of PDA nanoparticles induces a phase transition in the hydrogel, accelerating the drug release. The increased temperature disrupts the hydrogel network, enhancing the diffusion of DOX molecules. This dual response to pH and temperature ensures that the drug release is controlled and localized, particularly in the acidic tumor environment, where the hydrogel’s structure is more susceptible to thermal-induced changes. The pH and temperature-responsive drug release behavior highlight the hydrogel’s capacity to function as a smart delivery platform, wherein the localized lower pH and hyperthermia generated by NIR irradiation trigger the on-demand release of chemotherapeutic agents. As can be seen from Figure 3g, although hydrogel degradation had occurred to a certain extent within 3 days, DOX did not release significantly, because the diffusion of drugs was still controlled by the structure of the hydrogel network, and no obvious burst release occurred. Even under photothermal stimulation, the hydrogel did not completely degrade after a short time of laser irradiation (5 min), resulting in an increase in drug diffusion rate but still maintaining a controllable range. Such precision ensures minimal drug loss under physiological conditions, while efficiently delivering therapeutic doses to targeted tumor sites during photothermal activation. Compared to previous studies, the GPPD hydrogel demonstrated higher photothermal stability and enhanced drug retention under physiological conditions.

### 2.6. In Vitro Biocompatibility of Hydrogel

**Figure 4:**
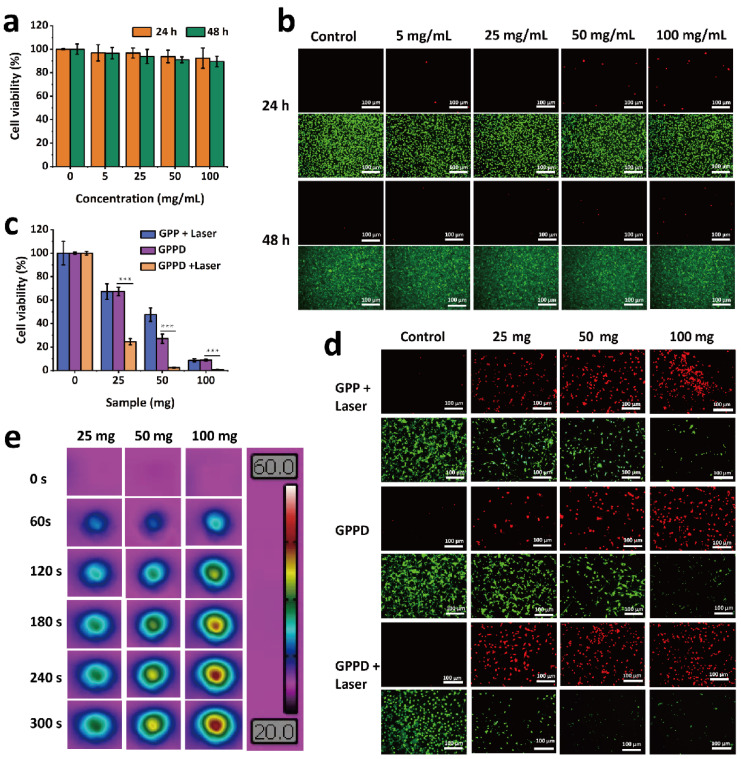
(**a**,**b**) Cytocompatibility experimental results of GPP hydrogel: (**a**) CCK-8 and (**b**) live/dead cell staining; (**c**,**d**) in vitro treatment results of GPP and GPPD hydrogel: CCK-8 (**c**) and live/dead cell staining (*** *p* < 0.001) (**d**); and (**e**) thermal imaging of GPPD hydrogel during in vitro photothermal treatment.

The biocompatibility of the hydrogel was systematically evaluated through hemolysis and cytocompatibility assays to ensure its safety for biomedical applications. As shown in Figure 3h, water-induced complete hemolysis served as the positive control, while PBS-treated samples represented the negative control. When red blood cells (RBCs) were exposed to GPPD hydrogels at concentrations of 5, 10, 25, and 50 mg/mL, the hemolysis rates remained consistently low, measuring 1.55 ± 0.39%, 1.68 ± 0.49%, 2.17 ± 0.57%, and 3.30 ± 0.60%, respectively. These values are well below the 5% hemolysis threshold established as a safety benchmark, confirming the hydrogel’s excellent blood compatibility and minimal erythrocyte damage potential. Further cytocompatibility (Figure 4a) study resulted in cell survival rates exceeding 85%, indicating negligible cytotoxicity. Additionally, live/dead cell staining provided visual confirmation of cell viability. Fluorescence microscopy (Figure 4b) revealed predominantly green fluorescent live cells across all treatment groups, with very few red fluorescent dead cells observed, further verifying the hydrogel’s non-cytotoxic nature. Collectively, these results demonstrate that the GPP hydrogel exhibits outstanding hemocompatibility and cytocompatibility, making it a safe candidate for biomedical applications, particularly in drug delivery and tissue engineering. The minimal hemolysis and high cell viability underscore the hydrogel’s potential for clinical use without inducing adverse effects on blood or cellular health.

### 2.7. In Vitro Tumor Treatment

In order to evaluate the killing ability of GPPD hydrogel on cancer cells, GPP and GPPD hydrogel extracts of different qualities (25, 50, and 100 mg) were incubated with mouse colon cancer cells (CT26) for 24 h and subjected to 808 nm irradiation. The in vitro therapeutic effect was evaluated by Cell counting kit-8 (CCK-8) detection and live/dead cell staining assay, respectively. In the gelation process, the initial concentration of DOX in GPPD hydrogel is 1 mg/mL.

As shown in Figure 4c, with the increase in the concentration of GPPD hydrogel extract, the cell survival rate showed a significant decline. Under photothermal conditions, the cell survival rate of GPP + Laser and GPPD + Laser groups decreased significantly with the increase in hydrogel content. At the same concentration, the survival rate of the GPPD + Laser group (*** *p* < 0.001 versus group GPPD) was significantly lower than that of the GPP + Laser group and the GPPD group. In addition, through the live/dead cell staining experiment (Figure 4d), it can be visually observed that with the increase in the concentration of the hydrogel extract, the number of live cells labeled with green fluorescence significantly decreases, while the number of dead cells labeled with red fluorescence gradually increases, while the number of live cells labeled with green fluorescence gradually decreases, and the fluorescence amount of green live cells in the GPPD + laser group is the least. The results showed that GPPD hydrogel showed an enhanced tumor cell-killing effect under photothermal stimulation.

At the same time, the thermal imaging map recorded during the experiment (Figure 4e) showed that the temperature of the hydrogel sample continued to rise with the extension of near-infrared irradiation time. This shows that GPPD hydrogels can efficiently absorb near-infrared light and convert it into heat. This significant rise in temperature has a direct effect on the killing of tumor cells, further enhancing the anti-tumor effect of the hydrogel.

These findings confirm that GPPD hydrogel possesses excellent photothermal conversion properties and effectively eradicates tumor cells under photothermal conditions, thereby providing substantial experimental support for its potential application in combined photothermal and chemotherapy. The versatility of this material positions it as a promising candidate for cancer treatment.

### 2.8. In Vivo Biological Safety

**Figure 5:**
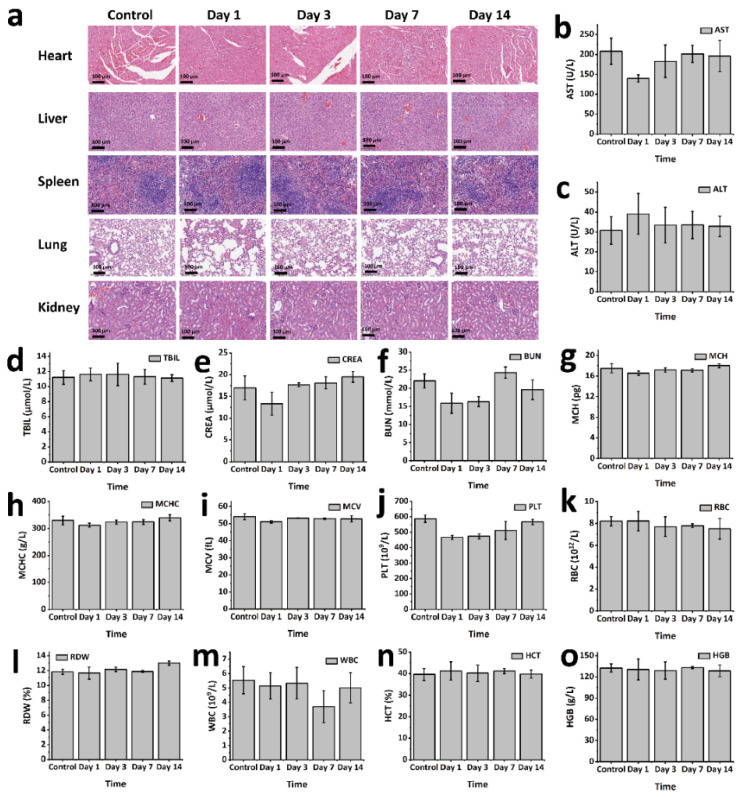
In vivo safety evaluation of GPP hydrogel: (**a**) H&E staining of major organs from implanted mice; (**b**–**f**) serum biochemical analysis results; and (**g**–**o**) blood routine analysis results.

To evaluate the in vivo biosafety of the developed hydrogel, comprehensive analyses were conducted using Kunming mice as the animal model. GPP hydrogel blocks (200 mg) were subcutaneously implanted into the dorsal regions of the mice. At designated time intervals (1, 3, 7, and 14 days post-implantation), the mice were sacrificed, and major organs—including the heart, liver, spleen, lungs, and kidneys—were harvested for histopathological analysis. H&E staining was performed to assess any potential morphological or pathological abnormalities induced by the hydrogel. As shown in the results (Figure 5a), no significant inflammation, tissue damage, or histological anomalies were observed across the organs when compared to the control group, confirming the excellent histocompatibility of the hydrogel. In addition to histological evaluation, biochemical and hematological parameters were monitored to assess the hydrogel’s systemic safety. Serum biochemical tests were performed to analyze key indicators of liver and kidney function, including aspartate aminotransferase (AST), alanine aminotransferase (ALT), total bilirubin (TBIL), creatinine (CREA), and urea nitrogen (BUN) (Figure 5b–f). All values remained within the normal physiological range, indicating that neither hepatic nor renal toxicity occurred as a result of hydrogel implantation. Finally, routine blood tests were conducted to evaluate potential systemic responses, such as inflammation, infection, or toxicity (Figure 5g–o). Parameters, including white blood cell count (WBC), hemoglobin (HGB), platelet count (PLT), red blood cell count (RBC), hematocrit (HCT), mean corpuscular volume (MCV), mean corpuscular hemoglobin (MCH), mean corpuscular hemoglobin concentration (MCHC), and red blood cell distribution width (RDW), exhibited no deviations from baseline values, further confirming the absence of systemic toxicity or adverse reactions. Collectively, these findings highlight the hydrogel’s excellent biocompatibility and biosafety profile. The absence of significant organ damage, systemic inflammation, or functional impairments indicates that the hydrogel is well-tolerated in vivo, underscoring its potential for clinical applications in tumor therapy.

### 2.9. In Vivo Tumor Treatment

**Figure 6:**
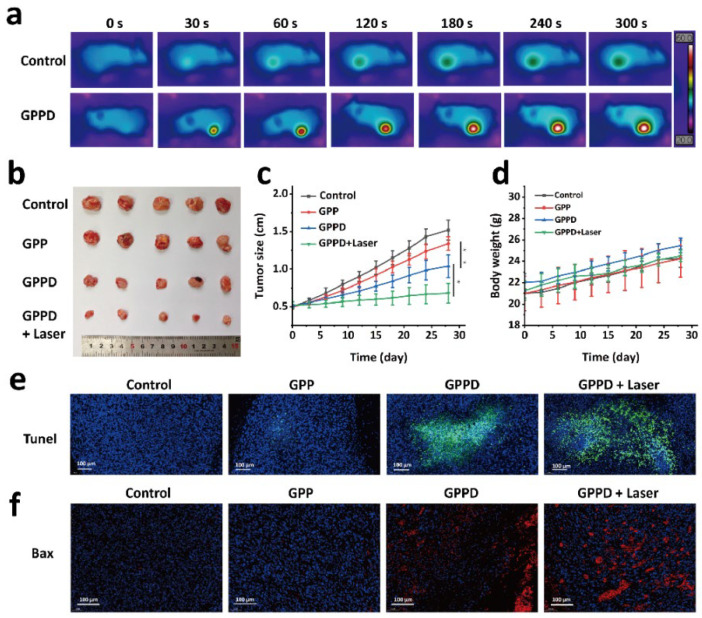
In vivo tumor treatment evaluation: (**a**) thermal imaging of nude mice during the in vivo tumor experiment; (**b**) physical appearance of tumors in each group at the end of the experiment; (**c**) tumor growth progression over the experimental period (* *p* < 0.05, ** *p* < 0.01); (**d**) changes in body weight of nude mice during the experiment; (**e**) TUNEL staining of tumors after treatment; and (**f**) Bax protein staining of tumors after treatment.

To evaluate the therapeutic efficacy of the GPPD hydrogel in a preclinical tumor model, CT26 colon cancer cells were subcutaneously inoculated into nude mice. The results revealed a significant and rapid temperature elevation in the tumor region of the GPPD + Laser group, exceeding 60 °C under NIR irradiation, while no substantial temperature changes were observed in the control groups (Figure 6a). This pronounced photothermal effect facilitated tumor ablation. Tumor size was monitored over 28 days, and the results demonstrated that tumors in the GPPD + Laser group exhibited remarkable growth inhibition, with nearly complete suppression observed by the study endpoint (82.3%). In comparison, tumors in the saline group exhibited aggressive growth, while partial suppression was observed in the GPP and GPPD-only groups. Figure 6b presents the final excised tumors, visually confirming the superior efficacy of the GPPD + Laser treatment. Quantitative tumor growth data (Figure 6c) indicated that the relative tumor volume in the GPPD + Laser group was significantly reduced (* *p* < 0.05 versus group GPPD) compared to all other groups (** p < 0.01, group GPP versus group GPPD). Importantly, body weight changes (Figure 6d) revealed no significant differences between treated and control groups, suggesting minimal systemic toxicity associated with the treatment. Histological analyses further corroborated the therapeutic effects of the hydrogel. TUNEL staining (Figure 6e) revealed extensive apoptotic cell death in tumors from the GPPD + Laser group, as indicated by strong green fluorescence. In contrast, the saline and GPP hydrogel groups displayed negligible levels of apoptosis. Bax protein immunohistochemistry (Figure 6f) demonstrated upregulated Bax expression, a key pro-apoptotic marker, in the GPPD + Laser group, indicating enhanced activation of apoptosis pathways. These results collectively highlight the remarkable potential of the GPPD hydrogel system for combined tumor therapy.

## 3. Conclusions

In this work, we successfully designed and synthesized a multifunctional hydrogel system combining γ-PGA, G5 dendrimers, and PDA nanoparticles to achieve combined PTT and controlled drug release for precision tumor treatment. The hydrogel exhibits excellent photothermal conversion properties under NIR irradiation, facilitating localized hyperthermia while triggering temperature-responsive drug release. In vitro studies demonstrate efficient photothermal-induced tumor cell ablation, enhanced drug delivery kinetics, and excellent hemocompatibility and cytocompatibility. In vivo evaluations further validate the hydrogel’s significant tumor suppression capabilities with minimal systemic toxicity and favorable biosafety, as evidenced by histopathological analysis and routine blood tests. This multifunctional hydrogel system not only demonstrates a high potential for clinical translation due to its biocompatibility and biodegradability but also provides a novel paradigm for integrating photothermal therapy and chemotherapy in cancer treatment. Unlike traditional inorganic photothermal agents, our γ-PGA-based hydrogel ensures efficient photothermal conversion while maintaining excellent in vivo degradability, reducing long-term toxicity concerns. Furthermore, our findings highlight that the temperature-responsive drug release mechanism can significantly enhance treatment efficacy while minimizing systemic side effects, paving the way for future applications in precision oncology.

## 4. Materials and Methods

### 4.1. Materials

All chemicals were used without further purification. G5 was purchased from Weihai Chenyuan Molecular New Materials Co., Ltd. (Weihai, China). γ-PGA was purchased from Shanghai Yika Biotechnology Co., Ltd. (Shanghai, China). Dopamine hydrochloride, doxorubicin hydrochloride, EDC, and NHS were purchased from Shanghai Aladdin Reagent Co., Ltd. (Shanghai, China). Sodium chloride was purchased from Greagent. Phosphate buffer was purchased from Sigma-Aldrich (Shanghai, China). CBS was purchased from Shanghai Titan Technology Co., Ltd. (Shanghai, China). Mouse fibroblasts and CT26 were purchased from Wuhan Pricella Biotechnology Co., Ltd. (Wuhan, China). Modified Eagle medium (DMEM) was purchased from Corning Incorporated (Shanghai, China). CCK-8 and live/dead cell staining kit were purchased from Shanghai Weiao Biotechnology Co., Ltd. (Shanghai, China). KM mice and BALB/c-Nude nude mice were purchased from GemPharmatech Co., Ltd. (Nanjing, China). The Institutional Review Board of Changhai Hospital Affiliated with Naval Medical University (SYXK (Shanghai) 2020–0033) approved the animal study protocol.

### 4.2. Synthesis of PDA Nanoparticles

To synthesize PDA nanoparticles, 1 g of G5 dendrimer was dissolved in 5 mL of deionized water to prepare a 200 mg/mL solution. Separately, 0.1 g of dopamine hydrochloride was dissolved in 10 mL of deionized water. To initiate the polymerization, 100 μL of the G5 solution was added to the dopamine solution under continuous stirring at room temperature. The reaction mixture was allowed to stir for 24 h, leading to the formation of PDA nanoparticles. The resulting suspension was dialyzed against deionized water for 3 days to remove unreacted dopamine and byproducts. The purified PDA nanoparticles were freeze-dried to obtain a powder. The morphology of the nanoparticles was characterized using scanning electron microscopy (SEM, Zeiss Sigma 300, Carl Zeiss AG, Oberkochen, Germany).

### 4.3. Preparation of Hydrogel

Hydrogels were synthesized by cross-linking γ-PGA with G5 dendrimer in the presence of EDC and NHS. First, 0.1 g of dopamine hydrochloride was dissolved in 8.9 mL of deionized water, followed by the addition of 100 μL of G5 solution. After stirring for 24 h, the PDA nanoparticle suspension was dialyzed against deionized water for 3 days to remove unreacted dopamine and byproducts. Then, 1 g of γ-PGA was added and thoroughly mixed to prepare the precursor solution. Separately, 0.1 g each of EDC and NHS were dissolved in 1 mL of deionized water to create an activator solution. The activator solution was quickly added to the precursor solution under vigorous stirring for 30 s. The mixture was then left undisturbed for 5 min to form the γ-PGA/G5/PDA composite hydrogel (GPP) hydrogel. For drug-loaded hydrogels, DOX was added to the above γ-PGA solution with a final concentration of 1 mg/mL. The other steps were identical to those described above. The final drug-loaded hydrogel was designated as γ-PGA/G5/PDA composite (GPPD) hydrogel. The same method was used to prepare GPPD with a DOX loading concentration of 50 mg/mL. The hydrogel samples were subjected to freeze-drying using a laboratory-grade freeze dryer to remove residual water while preserving the structural integrity. The dried hydrogels were then analyzed using SEM to examine their microstructural morphology.

### 4.4. Rheological Study

To investigate the dynamic changes of the hydrogel during the gelation process, a rotational rheometer (MARS III HAAKE, Thermo Fisher Scientific, Karlsruhe, Germany) was employed to evaluate its rheological properties. The experimental parameters were set as follows: temperature at 37 °C, a fixed oscillation frequency of 1 Hz, and a strain amplitude of 1%. A 200 μL aliquot of GPPD hydrogel precursor solution was placed onto a P20 TiL parallel plate rotor with a diameter of 20 mm and a gap of 1 mm, followed by the addition of 20 μL of activator solution. The storage modulus (G′) and loss modulus (G″) were recorded and analyzed using a time sweep test to monitor their evolution over the gelation period.

### 4.5. Swelling Behavior of Hydrogel

To evaluate the swelling performance of the hydrogel under different environmental conditions, experiments were conducted using saline to simulate the physiological environment and CBS buffer (pH = 5.4) to mimic the tumor microenvironment. Freeze-dried GPPD hydrogel samples of similar dimensions were initially weighed, and their dry weight was recorded as W0. Subsequently, the hydrogels were immersed in 20 mL of deionized water, saline, and CBS buffer (n = 3 for each condition) and incubated at 37 °C. At predetermined time intervals (0.5 h, 1 h, 2 h, 4 h, 8 h, 12 h, and 24 h), the hydrogels were removed, and the excess surface liquid was gently blotted using filter paper before weighing. The weight after water absorption was recorded as Wt. The swelling behavior was analyzed by calculating the swelling rate (%) of the hydrogel, using Equation (1). A swelling kinetic curve over 24 h was plotted to illustrate and compare the hydrogel’s swelling behavior and kinetics under different conditions.

(1) $$\mathrm{Swelling}\;\mathrm{rate}\;(\%)=\frac{W_{t}{-W}_{0}}{W_{t}}\times 100\%$$

### 4.6. In Vitro Photothermal Experiment

GPPD hydrogels of varying masses (12.5, 25, 50, and 100 mg) were placed in individual wells of a 96-well plate. Each hydrogel was irradiated with an 808 nm laser for 5 min at a power density of 1 W/cm^2^. Temperature changes during irradiation were recorded using an infrared thermal imaging camera. Deionized water (100 μL) was used as a blank control. To evaluate the impact of laser power, 50 mg of GPPD hydrogel was placed in a 96-well plate and irradiated with an 808 nm laser at power densities of 0.4, 0.8, and 1 W/cm^2^ for 5 min. The temperature changes were recorded using an infrared thermal imaging camera. For comparison, 50 μL of deionized water was used as a blank control. The photothermal behavior of the hydrogel in a saline environment was assessed by weighing 50 mg of GPPD hydrogel, placing it in a 96-well plate, and adding 100 μL of saline. The hydrogels were irradiated with an 808 nm laser at power densities of 0.4, 0.8, and 1 W/cm^2^ for 5 min. Temperature changes were monitored using an infrared thermal imaging camera. To evaluate the photothermal stability of the GPPD hydrogel, 50 mg of hydrogel was irradiated with an 808 nm laser at a power density of 1 W/cm^2^ for 5 min. The hydrogel was then allowed to cool naturally to room temperature. This heating–cooling cycle was repeated for six consecutive cycles, and temperature changes during each irradiation process were recorded using an infrared thermal imaging camera.

### 4.7. Drug Release

A specified amount of GPDD hydrogel was placed in a dialysis bag with a molecular weight cutoff of 3500 Da and immersed in 20 mL of saline or CBS buffer (pH 5.4) (n = 3 for each condition). The dialysis bags were incubated in a shaker at 37 °C with an agitation speed of 120 rpm. At predefined time intervals (0.5 h, 1 h, 2 h, 4 h, 8 h, 12 h, 24 h, 48 h, and 72 h), 1 mL of the supernatant was collected, and an equal volume of fresh corresponding buffer was added to maintain the total volume. The absorbance of the collected supernatant was measured at 480 nm using a UV-visible spectrophotometer (Shimadzu UV-3600, Shimadzu Corporation, Kyoto, Japan), and the cumulative release efficiency of DOX was calculated based on a pre-established standard calibration curve. To assess the influence of photothermal conditions on DOX release, additional samples from the CBS buffer group (n = 3) were subjected to photothermal stimulation. After 24 h of incubation under the same conditions, the samples were exposed to 50 °C water. The release efficiency of DOX was subsequently determined.

### 4.8. In Vitro Degradation of Hydrogel

The in vitro degradation performance of the GPPD hydrogel was evaluated by measuring the residual mass ratio over time under different conditions. Freeze-dried hydrogel samples were prepared, and their initial weight was recorded as W0. The samples were fully immersed in 10 mL of deionized water, saline, or CBS buffer (pH 5.4) and incubated at 37 °C in a constant temperature environment (n = 3 for each condition). At predetermined time points (1 day, 2 days, 3 days, 5 days, 7 days, 10 days, and 14 days), the hydrogels were retrieved from the solution and gently rinsed three times with deionized water to remove surface residues. The samples were then freeze-dried and weighed as Wt. The degradation behavior was analyzed by calculating the residual mass ratio (%) of the hydrogel using Equation (2):

(2) $$\mathrm{Residual}\;\mathrm{mass}\;\mathrm{ratio}\;(\%)=\frac{W_{t}}{W_{0}}\times 100\%$$

### 4.9. In Vitro Blood Compatibility

The hemolytic performance of the GPPD hydrogel was evaluated using Kunming mouse whole blood. A total of 2 mL of whole blood was collected and centrifuged at 5000 rpm for 5 min to remove the plasma. The RBCs were washed three times with PBS until the residual plasma was completely removed. The washed RBCs were resuspended in 50 mL of PBS for further use. The experimental groups consisted of 7.5 mg, 15 mg, 37.5 mg, and 75 mg of GPPD hydrogel (n = 3 for each concentration) mixed with 1.5 mL of PBS, followed by the addition of 0.3 mL of the RBCs suspension. The negative control group consisted of 1.5 mL PBS and 0.3 mL of the RBCs suspension, while the positive control group contained 1.5 mL of deionized water and 0.3 mL of the RBCs suspension. All samples were incubated in a 37 °C incubator for 2 h. After incubation, all samples were centrifuged at 5000 rpm for 5 min, and the supernatant was carefully collected. The absorbance of the supernatant at 541 nm was measured using a UV-visible spectrophotometer. The hemolysis ratio (%) was calculated using Equation (3):

(3) $$\mathrm{Hemolysis}\;\mathrm{ratio}\;(\%)\;=\frac{(\mathrm{Ds}-\mathrm{Dn})}{\left(\mathrm{Dp}-\mathrm{Dn}\right)}\times 100\%$$

Ds is the absorbance of the supernatant of the experimental group, Dn is the absorbance of the negative control group, and Dp is the absorbance of the positive control group.

### 4.10. In Vitro Cytocompatibility

The cytocompatibility of the GPP hydrogel was assessed using the CCK-8 assay and live/dead cell staining. Mouse fibroblast cells were seeded into 96-well plates at a density of 1 × 10^4^ cells per well and incubated at 37 °C in a humidified atmosphere containing 5% CO_2_ for 12 h to allow for cell adhesion. The GPP hydrogel was sterilized, and hydrogel extracts were prepared using DMEM at the GPP hydrogel concentrations of 0, 5, 25, 50, and 100 mg/mL. After removing the supernatant from each well, 100 μL of hydrogel extract at the corresponding concentration was added (n = 3 per group). DMEM alone was used as the blank control. The cells were cultured for 24 and 48 h. Following the incubation, the cells were washed three times with PBS to remove residual hydrogel extract. Cell viability was assessed using the CCK-8 assay kit (Dojindo Laboratories, Kumamoto, Japan), with absorbance measured at 450 nm. The relative cell viability under different hydrogel concentrations and incubation times was calculated to evaluate the cytotoxicity of the GPP hydrogel. Subsequently, the cells were stained using Calcein-AM (green fluorescence for live cells) and propidium iodide (PI, red fluorescence for dead cells) following the manufacturer’s instructions. The stained cells were observed and imaged using an inverted phase contrast fluorescence microscope (MARS III HAAKE) to evaluate the hydrogel’s cytotoxicity.

### 4.11. In Vitro Tumor Treatment Experiments

To evaluate the therapeutic effect of GPP and GPPD hydrogels on tumor cells, mouse CT26 cells were inoculated into 96-well plates with a cell density of 1 × 10^4^ cells per well, incubated at 37 °C, 5% CO_2_ environment for 12 h, and discarded the supernatant. After sterilization, GPP and GPPD hydrogels were directly added into 96-well plates with hydrogels of 0, 25, 50, and 100 mg per well (n = 3). Then, an 808 nm laser was used to illuminate the perforating plates for 5 min with a laser power of 1 W/cm^2^. After irradiation, the culture continued for 24 h. A set of GPPD hydrogels (0, 25, 50, and 100 mg (n = 3)) were obtained and incubated directly with cells in 96-well plates for 24 h. The hydrogel extract was discarded, the cells were washed with PBS three times, the cell survival rate was calculated using CCK-8, and the cells were stained by live/dead staining. The appearance of live and dead cells was observed and photographed using an inverted phase contrast microscope, and the effect of GPP and GPPD hydrogels on tumor cells in vitro was evaluated.

### 4.12. In Vivo Degradation

To investigate the in vivo degradation of GPP hydrogel, a degradation study was conducted in KM mice. A total of 200 mg of GPP hydrogel was subcutaneously implanted into the dorsal region of the mice (n = 3). At predetermined time points (1, 3, 5, 7, and 10 days), the hydrogel samples were carefully retrieved, cleaned, and weighed. The percentage of mass loss over time was calculated to evaluate the in vivo degradation profile of the hydrogel.

### 4.13. In Vivo Biosafety

To evaluate the in vivo biosafety of GPP hydrogel, an animal model using KM mice was employed. GPP hydrogel blocks (0.2 g) were subcutaneously implanted into the dorsal region of the mice (n = 3). Mice were sacrificed at predetermined time points (1, 3, 7, and 14 days post-implantation). The main organs were harvested for histological analysis through hematoxylin and eosin (H&E) staining. Tissue sections were observed and imaged using an inverted phase contrast microscope to assess any histopathological changes induced by the hydrogel. Additionally, blood samples were collected for both serum biochemistry and routine blood analysis. Serum biochemical parameters were measured using the Beckman Coulter Unicel DxC 800 fully automated biochemical analyzer (Beckman Coulter, Inc., Brea, CA, USA). Routine blood tests were conducted using the Sysmex XS-800i fully automated blood analyzer (Sysmex Corporation, Kobe, Japan).

### 4.14. In Vivo Tumor Treatment Experiments

CT26 tumor cells were subcutaneously inoculated into the flanks of nude mice. When the tumor volume reached approximately 0.5 cm in diameter, the mice were randomly assigned to four treatment groups, with six mice per group. The mice were anesthetized before receiving the following treatments: Group 1 was injected with 200 mg of GPP hydrogel; Group 2 was injected with 200 mg of GPPD hydrogel; Group 3 received 200 mg of GPPD hydrogel and underwent laser irradiation (808 nm NIR laser, 1 W/cm^2^, 5 min); and Group 4 was injected with 200 μL of saline (control group). Throughout the experiment, the initial body weight and tumor volume of each mouse were recorded, and images were taken for visual tracking. Tumor growth was monitored at time points 0, 3, 6, 9, 12, 15, 18, 21, 24, and 28 days. Key evaluation parameters included relative tumor volume (V/V_0_, where V_0_ represents the tumor volume at day 0), changes in body weight, and the visual appearance of the tumors. After the study, tumors were excised and subjected to TUNEL staining and Bax protein immunohistochemistry to assess the impact of the various treatments on tumor cell apoptosis.

### 4.15. Statistical Analysis

All data were represented as mean ± standard deviation (SD). One-way ANOVA was used to evaluate comparisons between several groups. A *p*-value below 0.05 was deemed statistically significant (* *p* < 0.05, ** *p* < 0.01, *** *p* < 0.001).

## Data Availability

The data presented in this study are available on request.
